# Digital PCR: A Reliable Tool for Analyzing and Monitoring Hematologic Malignancies

**DOI:** 10.3390/ijms21093141

**Published:** 2020-04-29

**Authors:** Nicoletta Coccaro, Giuseppina Tota, Luisa Anelli, Antonella Zagaria, Giorgina Specchia, Francesco Albano

**Affiliations:** Department of Emergency and Organ Transplantation (D.E.T.O.), Hematology Section, University of Bari, 70124 Bari, Italy; nicoletta.coccaro@uniba.it (N.C.); giuseppina.tota@uniba.it (G.T.); luisa.anelli@uniba.it (L.A.); antonellazagaria@hotmail.com (A.Z.); specchiagiorgina@gmail.com (G.S.)

**Keywords:** digital PCR, dPCR, next-generation sequencing, NGS, hematology, somatic mutation, minimal residual disease, MRD monitoring

## Abstract

The digital polymerase chain reaction (dPCR) is considered to be the third-generation polymerase chain reaction (PCR), as it yields direct, absolute and precise measures of target sequences. dPCR has proven particularly useful for the accurate detection and quantification of low-abundance nucleic acids, highlighting its advantages in cancer diagnosis and in predicting recurrence and monitoring minimal residual disease, mostly coupled with next generation sequencing. In the last few years, a series of studies have employed dPCR for the analysis of hematologic malignancies. In this review, we will summarize these findings, attempting to focus on the potential future perspectives of the application of this promising technology.

## 1. Introduction

The introduction of the digital polymerase chain reaction (dPCR) in cancer research is quite recent. The history of this third-generation polymerase chain reaction (PCR) technology started in the 1990s with the first attempts to obtain single PCR molecules, applying limiting dilution conditions [1,2,3,4,5,6]. In 1992, Sykes et al. first explored the idea of using limiting dilution, PCR and Poisson statistics to quantitate the rearranged immunoglobulin heavy chain (*IgH*) gene derived from a leukemic clone in a background of excess rearranged *IgH* genes from normal cells [6]. At the end of the decade, the introduction of emulsion-based formulations for sub-partitioning and of nanofluidics, as well as the expansion of software tools, allowed for the development of more performant instruments able to subdivide the reaction in very small volume partitions [7]. The technology, as we know it today, owes its name to Vogelstein et al. [8,9], who were the first to apply dPCR platforms in the oncologic field [8]. Since then, different systems have been invented, such as the microfluidic chamber-based BioMark Digital PCR from Fluidigm [10], the chip-based Quantstudio 12k/3D dPCR System from Thermo Fisher Scientific [11], the droplet-based QX-100/QX-200 Droplet Digital PCR (ddPCR) Systems from Bio-Rad Laboratories [12], the RainDrop dPCR from RainDance Technologies [11], the Crystal dPCR System with the Naica System from Stilla Technologies [13], the Clarity dPCR system from JN MedSys [14], and FORMULATRIX dPCR from QIAGEN. Each variation of the dPCR methodology has been demonstrated to be useful for studying cancer, yielding comparable results in regards to nucleic acid quantification, sensitivity and specificity [15,16]; however, of them all, ddPCR seems to present greater diffusion as compared to the other technologies, likely because of its qualities as regards ease of use and application, and adaptability, saving time and effort. It is the digital platform most commonly used for cancer applications [17], and also in studies on hematologic malignancies [18,19,20,21].

An in-depth study of the technical details is not the within the scope of this manuscript, and for this type of examination we cite more specialized reviews [22,23]. In general, dPCR is based on the principle of partitioning the sample into several PCR sub-reactions containing single, few or no target-sequences; next, PCR partitions are read and counted as negative or positive by thresholding based on their fluorescence amplitude; then the number of positive and negative partitions is used to calculate the concentration of the target sequence, applying an analysis method based on Poisson’s statistics [24,25]. These kind of statistics correlate the efficiency of the partitioning of PCR reactions with the sensitivity, linking the theoretical depth of analysis to the number of compartments generated [26]. In the case of ddPCR, the partitioning into thousands of nanoliter droplets occurs, generated by mixing the sample in a water-in-oil emulsion [27]. By means of this approach, dPCR allows the absolute quantification of target nucleic acids in a sample, without the need of calibrators and standard curves, solving some shortcomings of Real-time Quantitative PCR (qPCR) [6,8,28]. Indeed, compartmentalization renders PCR less sensitive to reaction inhibitors, and reduces any template competition, allowing for the detection of rare target sequences in a wild-type background. In order to establish robustness, for each dPCR, assay Limit of Blank (LoB), Limit of Detection (LoD), and Limit of Quantitation (LoQ) have to be determined, where LoB is defined as the highest apparent target concentration likely found when replicates of a blank sample containing no target sequences are analysed; LoD is the lowest target concentration expected to be distinguished from the LoB and at which detection is feasible; and LoQ is the lowest concentration at which the target can be quantified [29]. These parameters define the quality of a dPCR test. In view of the high precision and ultrahigh sensitivity obtainable (mutated allele frequency detected down to the 0.001% level), dPCR is suitable for Minimal Residual Disease (MRD) monitoring approaches [30,31]. These characteristics make dPCR a technology with great potential as regards sensitivity, specificity and accuracy, and for these reasons it has been employed in several studies of various types of hematologic diseases.

In this literature review we summarize the results obtained from research applying dPCR, and mostly ddPCR, in the field of onco-hematology. In the conclusion, we attempt to envisage what the future of this technology for the study of hematologic malignancies may be, highlighting its strengths and disadvantages, in order to see if there could be further applicability spaces in research and in clinical practice.

## 2. dPCR for Detecting Somatic Mutations

**Figure 1:**
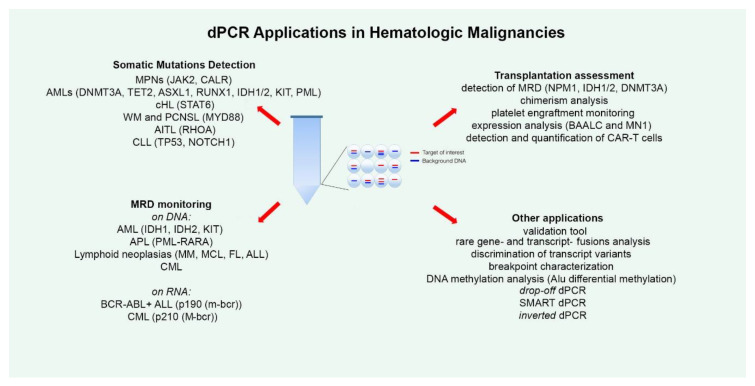
The figure summarizes all the current applications of droplet digital polymerase chain reaction (dPCR) technology explored in the field of hematology. For each disease, the studied alterations are indicated in round brackets. MPNs—Myeloproliferative Neoplasms; AML—Acute Myeloid Leukemias; cHL—Classical Hodgkin Lymphoma; WM—Waldenström Macroglobulinemia; PCNSL—Primary Central Nervous System Lymphomas; AITL—Angioimmunoblastic T-Cell Lymphoma; CLL—Chronic Lymphocytic Leukemia; MRD—Minimal Residual Disease; APL—Acute Promyelocytic Leukemia; MM—Multiple Myeloma; MCL—Mantle Cell Lymphoma; FL—Follicular Lymphoma; ALL—Acute Lymphoblastic Leukemia; CML—Chronic Myeloid Leukemia; SMART-ddPCR—Somatic Mutation Allelic Ratio Test-ddPCR.

dPCR has been applied for the detection of several somatic mutations, both for absolute allele quantification and for rare mutation detection (Figure 1). The most numerous studies in this regard have been conducted on Philadelphia negative (Ph-) chronic Myeloproliferative Neoplasms (MPNs), such as polycythemia vera (PV), essential thrombocythemia (ET) and myelofibrosis (MF). Ph- MPNs are associated with driver genes mutations, like *JAK2* in nearly 90% of PV patients and in around 50% of all ET and MF patients [32], and *CALR*, present in about 20%–35% of patients affected by ET and primary MF [33]. Gene expression dysregulation due to chromosomal rearrangements has rarely been reported in Ph- MPNs [34]. The first report dates back to 2015, when Fontanelli et al. compared qPCR and ddPCR in detecting the *JAK2^V617F^* mutation, the major MPN diagnostic criterion [35]. They identified 225 MPN patients presenting the *JAK2^V617F^* mutation by conventional qPCR and 99 of these were evaluated also by ddPCR. The specificity was absolutely concordant between the two methods, but the sensitivity was half a log higher for ddPCR than qPCR [35]. Similar conclusions were obtained in other subsequent studies, allowing researchers to state that ddPCR is a very suitable, precise, and sensitive method for the quantification of the *JAK2^V617F^* mutation, allowing a sensitivity of 0.01%, equitable with that achieved by Kröger et al. with an ARMS qPCR in 2007 [32,36,37,38]. Moreover, Nystrand et al. explored the use of ddPCR to test if serum is a good material for the detection and quantification of the *JAK2^V617F^* mutation. At first, the *JAK2* mutation was detected in peripheral blood (PB) samples of 47 patients, and afterwards, among these, 45 of 47 corresponding serum samples showed a very strong correlation between PB and serum. The overall detection sensitivity was 96% and the mutation was detected in all cases where the mutant allele load was above 1%. The observation of a significantly higher allele burden detected in serum compared to PB highlighted the point that ddPCR could be a reliable method for detecting the *JAK2^V617F^* mutation also in serum [39].

dPCR was tested also for detection of the *CALR* gene mutations. In 2016, Mensier et al. and Badbaran et al., concomitantly performed ddPCR for the absolute quantification of *CALR* type 1 and 2 mutations, demonstrating developing ddPCR assays that were able to reach a sensitivity of 0.02%. In the same year, Anelli et al. also described a ddPCR assay for the *CALR* type 1 and 2 mutation quantification [40]. They described a LOD of 0.01% and a sensitivity of 0.1% during patient follow-up (FU), analyzing 10 ng of DNA per sample [40]. Although the value of the *CALR* allelic burden has not yet been established at the disease onset, these reports laid the foundation for ddPCR utility as a reliable method for *CALR* mutations MRD monitoring [41,42].

Acute Myeloid Leukemias (AMLs) instead, are the most common and severe form of acute leukemia diagnosed in adults. Owing to its heterogeneity, AML is divided into classes associated with different treatment outcomes, disease markers and specific gene expression profiles. AML, unfortunately, frequently relapses after complete remission (CR), and so improved detection and phenotypic characterization of treatment-resistant residual leukemic cells are urgently needed [43]. Parkin et al. employed ddPCR to study variant allele fractions (VAFs) of frequently mutated genes in samples from AML patients in CR, to evaluate the persistence of mutated clones at a level as low as 0.002%. They showed that most of the AML cases showed a residual abnormal oligoclonal hematopoiesis, very rare cells, as few as 1 in 15,000, being genomically comparable to the principal blast populations at diagnosis and being totally clonally present at relapse. They are, therefore, a common source of AML relapse. Because they observed that overall survival (OS) was associated with the mutant allele burden in AML, their data restricted the list of gene mutations, finally including *DNMT3A*, *TET2*, *ASXL1*, *RUNX1*, and *IDH1/2*, shown to be useful in MRD-based prognostication in AML [44]. Instead, Tan and colleagues employed both Sanger sequencing (SS) and ddPCR to analyze the clone composition and dynamic evolution of *KIT* double gene mutations in core binding factor (CBF)-AML patients, revealing that the double mutations can occur in either the same or different clones and the latter may generate a different sensitivity to CBF-AML treatment [45]. Lastly, a study by Alfonso and colleagues used a ddPCR assay for *PML^A216V^* gene mutation detection and quantification, linked to trioxide arsenic therapy resistance in acute promyelocytic leukemia (APL) patients. They firstly identified the *PML^A216V^* mutation by ddPCR in 5 of 13 APL patients, and then extended the screening to all evaluable serial FU samples, detecting the mutation in all patients showing treatment resistance. They assumed that it may have been present at subclonal level before therapy and then later selected under treatment pressure. These data suggest that ddPCR can be successfully recruited for *PML^A216V^* gene mutation screening and that this procedure may allow for the detection of mutant cases earlier than conventional sequencing [46].

dPCR has been applied also in studies of lymphomas. Classical Hodgkin lymphoma (cHL) accounts for 30% of all lymphomas [47]; in the last few years, a series of Next-Generation Sequencing (NGS) studies highlighted the involvement of a series of recurrent somatic mutations [48,49,50,51,52,53,54,55,56,57,58]. However, a common challenge that is an obstacle to the detection of somatic mutations is the paucity of Hodgkin and Reed–Sternberg (HRS) cells in biopsy samples (0.1% to 10% of total tumor tissue) [47], so that previous microdissection seems to be mandatory. For this reason, the possibility of performing liquid biopsy with highly sensitive detection methods such as NGS and ddPCR could pave the way for a real improvement in cHL management [50,59]. Recently, NGS and ddPCR have been applied to determine whether the pattern of acquired mutations detected in the tissue biopsy DNA of 24 cHL biopsies can also be observed in cell-free DNA (cfDNA) at the time of diagnosis [60]. Bessi et al. observed similar or partially similar patterns of mutations in about one third of cHL cases, and at least one mutation in tissue biopsy DNA and/or cfDNA in 70% of cases. Indeed, they designed a ddPCR assay for the N417Y mutation of the *STAT6* gene, the most commonly observed variant, and used it for MRD monitoring [60].

For the diagnosis of Waldenström Macroglobulinemia (WM), bone marrow (BM) biopsy seems to be mandatory, to define the infiltration of indolent lymphoplasmacytic lymphoma (LPL) (i.e., monoclonal lymphocytes, lymphoplasmacytes and plasma cells (PC) in BM) and monoclonal IgM protein secretion [61,62]. However, the differential diagnosis can sometimes be troublesome both at the morphologic and the immunophenotypic level. The *MYD88^L265P^* mutation is a hallmark of WM, and its finding can be conclusive for the diagnosis [63,64]. In 2018, Drandi et al. demonstrated the feasibility of ddPCR for the detection and MRD monitoring of the *MYD88^L265P^* mutation in WM in different tissues (BM, PB and cfDNA) [65]. They reported a sensitivity of the ddPCR assay of 5.00 × 10^−5^, far superior to the classical PCR method, after analyzing 291 samples (194 baseline samples and 97 FUs) from 148 patients (133 with WM, 11 with IgG lymphoplasmacytic lymphoma and four with IgM monoclonal gammopathy of undetermined significance). About 95% of BM and 71% of PB baseline samples were positive for *MYD88^L265P^* [65]. The FU analysis was compared with IGH-based MRD analysis in 10 patients, and they observed that the two methods were equivalent. Lastly, the analyses of plasma ctDNA from 60 patients were concordant with BM data, concluding that BM, PB, as well as ctDNA, are informative in treatment-naïve patients for *MYD88^L265P^* detection in WM; instead in relapsed treated patients, where BM analysis should be preferred, considering that PB has a one log lower median mutated:WT ratio compared to BM, ddPCR analysis on plasma ctDNA could represent a promising, less invasive alternative to BM [65]. These results were corroborated in a more recent report by Lo Schirico et al., demonstrating the useful applications of this ddPCR assay in daily practice [65,66].

The *MYD88^L265^* gene mutation has been demonstrated to be a diagnostic and prognostic marker also in more than 70% of primary central nervous system lymphomas (PCNSL) [67]. A 2017 study by Hattori et al. tested ddPCR and targeted deep sequencing (TDS) techniques for *MYD88^L265P^* detection in PCNSL patients in diagnostic paired tumor-derived DNA and cfDNA samples. They concluded that ddPCR could be employed for non-invasive diagnosis on the liquid biopsy of cfDNA, but not for MRD monitoring in PCNSL [67]. In a similar work, Zorofchian et al. applied again ddPCR to detect *MYD88* mutations (*L265P* and *V217F*) in isolated cerebrospinal fluid (CSF)-circulating tumor DNA (ctDNA), in conjunction with evaluating the patient-matched central nervous system (CNS) lymphoma tissue [68]. The *MYD88^L265P^* mutation was detected both in formalin-fixed paraffin-embedded (FFPE) tissue from the brain biopsy and in the CSF-ctDNA, highlighting that CSF ctDNA analysis through ddPCR could be a potential minimally invasive approach to diagnosing patients with suspected CNS lymphomas [68].

Tanzima Nuhat et al., in a 2018 study, compared NGS, ddPCR and the peptide nucleic acid-locked nucleic acid (PNA-LNA) clamp method to find which was most efficacious for detecting the *RHOA*^G17V^ gene mutation, an essential element for the appropriate diagnosis of angioimmunoblastic T-cell lymphoma (AITL), a subtype of nodal peripheral T-cell lymphoma (PTCL) [69]. In these analyses, NGS identified the *RHOA*^G17V^ mutation in 27 of 67 (40.3%) PTCL samples, while ddPCR and the PNA-LNA clamp method both detected the *G17V* mutation in 31 of 67. Indeed, VAFs calculated with ddPCR and NGS were highly concordant. However, NGS analysis found three other RHOA mutations involving position *G17*, whose presence could be detected but not appropriately defined with the ddPCR and the PNA-LNA clamp methods, because they showed abnormal signal patterns, different from those of *G17V* mutated and wild type alleles. The authors concluded that a combination of ddPCR/PNA-LNA clamp methods and NGS is the best way to identify *RHOA*^G17^ substitutions and to support the diagnosis of AITL [69].

Few studies have been performed on Chronic Lymphocytic Leukemia (CLL) testing dPCR assays. CLL is the most common leukemia in adults, characterized by an extremely heterogeneous clinical course, linked to immunogenetic markers associated with different disease prognoses [70]. The first report was made in 2016 by Amin et al., who employed ddPCR or deep re-sequencing to calculate the VAFs of 19 genes during relapse, identifying, in *TP53* mutations, the dominant subclonal gene driver of relapse, and revealing uncommon mutations in *ATP10A*, *FAT3*, *FAM50A* and *MGA* with increased relapse-associated VAFs. In contrast, VAFs of mutations in *NOTCH1*, *SF3B1*, *POT1*, *FBXW7*, *MYD88*, *NXF1*, *XPO1*, *ZMYM3* or *CHD2* seemed not to vary at the moment of relapse [71].

Minervini et al. carried out a study investigating 88 CLL patients at diagnosis employing a *c.7541-7542delCT NOTCH1* mutation ddPCR assay, and also analyzing the *NOTCH1mut* allelic burden, indicated as fractional abundance (FA). They then monitored the FA variation over time in 10 cases. Our data revealed that with the ddPCR method, the incidence of *NOTCH1mut* in CLL was much higher (53.4%) than previously reported; indeed, in FU analysis, a statistically significant decrease of the *NOTCH1mut* FA was found from diagnosis after treatment. This same was noted in the relapsed samples as compared to complete or partial remission samples, suggesting that screening by ddPCR might help to identify patients in need of an earlier clinical FU during the “watch and wait” period, and after standard chemotherapy [72]. 

## 3. dPCR for MRD Monitoring

A surely important dPCR application area is disease monitoring during patients’ FU to track tumor-associated mutations or mutations arising secondarily in response to and selected by therapy, indicating a drug resistance mechanism, in order to monitor disease progression (Figure 1).

Several studies employed dPCR for MRD monitoring in AML. The first report dates back to 2014, when Bacher et al. used dPCR (Fluidigm) to quantify very rare mutations (*non-A, -B, -D*) in *NPM1* gene, at diagnosis and during FU for MRD monitoring. The dPCR sensitivity was comparable to that of conventional qPCR and enabled MRD diagnostics in all rare *NPM1* mutation subtypes tested [73]. Petrova et al. used mutations in the *IDH1* and *IDH2* genes as an MRD indicator to monitor 90 AML patients through both massive parallel sequencing and ddPCR. The study showed that both technologies were appropriate for MRD monitoring, although in some cases ddPCR presented a higher sensitivity [74]. *IDH1/2* mutations were also screened in AML patients by Grassi at al. using a new method, the drop-off ddPCR, described below [75]. The assay reached a sensitivity of 2 × 10^−3^; the researchers screened 60 AML patients, identifying at diagnosis 11 *IDH2*-mutated cases with a median mutational burden of 13.7%. Parallel SS did not identify mutations in 5/11 cases, likely because of the low mutational burden [75]. The Liu research group, instead, explored the use of the *KIT* mutation as MRD indicator in CBF-AML. They analyzed 20 CBF-AML patients with ddPCR, noting that the *KIT* gene mutation level during CR in the relapsed patients cluster was statistically significantly higher than that in the remission group. They also monitored the *KIT* gene mutation level in 10 patients from the initial diagnosis to CR, and found that the degree of remission was significantly lower in patients without recurrence than in patients with recurrence. These data showed that the *KIT* gene mutation is a helpful marker for MRD monitoring in CBF-AML, and that ddPCR is an effective method for this purpose [76].

In APL, qualitative PCR and qPCR are two well-established techniques for MRD monitoring. Despite their considerable sensitivity and specificity, both methods have intrinsic limitations, such as in qualitative MRD assessment and relative quantification, especially when MRD levels oscillate under the limit of detection of qPCR and are detectable by qualitative nested PCR. The first study to apply ddPCR for the absolute quantification of the *PML-RARA* transcript was performed by Albano et al. [77], and later, in a following study, the same group also tested ddPCR to monitor MRD in 21 APL patients [78]. The results obtained from ddPCR exhibited a good linearity, a great efficiency and a sensitivity similar to PCR and qPCR. On primary samples, ddPCR showed a sensitivity and specificity of 95% and 91% for the bcr1 and bcr3 transcripts, respectively, offering the advantage of absolute quantification [78]. These data are in agreement with those of Yuan et al. and for these reasons, ddPCR can be regarded as a good potential alternative technique for quantifying *PML-RARA*, particularly during molecular FU of patients at high risk of relapse [78,79]. 

MRD assessment is considered to provide valuable prognostic information in various lymphoid malignancies, and is frequently included as a secondary goal in clinical trials [80,81,82,83,84]. The application of dPCR for MRD monitoring of Multiple Myeloma (MM), Mantle Cell Lymphoma (MCL), and Follicular Lymphoma (FL) has been explored in several reports, that compared the results with qPCR [85,86,87,88]. All the studies reported a good concordance between the two techniques [85,86,87,88]; indeed, ddPCR was demonstrated to be successful in 100% of analyzed cases, whereas qPCR failed in generating reliable results for three cases, because unable to produce adequate standard curves [85]. In the work by Della Starza et al., which summarized the collaborative efforts of four laboratories belonging to the Fondazione Italiana Linfomi (FIL) MRD Network for FL and MCL MRD assessment comparing PCR and qPCR [87], it was demonstrated that borderline samples that resulted alternatively positive and negative regardless of the method and the type of rearrangement showed no interlaboratory discordance at dPCR analysis. Thus, dPCR is a valid alternative method for the analysis of samples with the lowest MRD levels, which pose a challenge for interlaboratory reproducibility [87]. Furthermore, FL patients with undetectable/low levels (<10^−5^) of circulating *BCL2/IGH+* cells at diagnosis or resulting persistently MRD negative during FU, exhibited a significantly better progression-free survival (PFS) when analyzed by ddPCR [86,88]. Very recently, Drandi et al. compared ddPCR with qPCR in 416 MCL MRD samples, 61% of which fell below the quantitative range of the standard curve. Additionally, this analysis showed comparable results for ddPCR and qPCR, particularly for samples with at least 0.01% positivity; whereas ddPCR was shown to be preferable to qPCR for low-level positivity samples, since for such analyses, it provided more robust quantification [89].

Chronic myeloid leukemia (CML) is a myeloproliferative neoplasm characterized by the t(9;22) translocation, which causes the formation of the chimeric *BCR-ABL1* fusion gene. This fusion gene is then translated into a BCR-ABL1 oncoprotein that triggers neoplastic transformation of the hematopoietic stem cell. CML is one of the first malignancies for which the principle of targeted therapy has been successfully applied thanks to the use of tyrosine kinase inhibitors (TKI) [90,91]. The Europe Against Cancer (EAC) qPCR assay has some inherent limitations, such as the low accuracy at the lower end of the calibration curve, as well as inter-laboratory discrepancies in assay execution. The use of conversion factors and the introduction of the International Scale (IS) has reduced but not eliminated these issues [92]. To overcome these limitations, various studies have aimed to highlight the strengths of dPCR compared to qPCR, also proposing possible improvements of the method. Maier et al., for example, optimized a duplex ddPCR, which, thanks to improved settings, increased *ABL1* and *BCR-ABL1* fluorescence signals two- and five-fold, respectively, and upgraded the resolution between positive and negative drops [93]. Franke et al., instead, created a duplex EAC *BCR-ABL1* ddPCR assay, that yielded comparable *ABL1* and *BCR-ABL1* transcript levels to those obtained by qPCR, but showed a switch to a lower molecular response category compared to qPCR adapted to IS [94]. Lastly, Chung et al. evaluated the first commercially available ddPCR-based in vitro diagnostics test, the QXDx *BCR-ABL1* %IS (Bio-Rad, Hercules, CA, USA) ddPCR assay. The limit of blanks, limit of detection, and limit of quantification claimed by the manufacturer were confirmed, and the two methods, the QXDx *BCR-ABL1* %IS ddPCR assay and the Ipsogen *BCR-ABL1* Mbcr IS- qPCR assay, showed a very high correlation (r=0.996) [95]. 

Furthermore, some studies have been carried out to evaluate MRD using dPCR in CML patients. The first dates back to 2011, when Goh et al. compared qPCR with dPCR for this purpose. They screened 62 samples from 43 patients with CML by conventional qPCR, and the analyses were replicated with qPCR (r-qPCR) and/or nanofluidic dPCR (BioMark Digital PCR, Fluidigm). They confirmed the correlation of dPCR with conventional qPCR using samples with various MRD levels. The addition of pre-amplification to r-qPCR and dPCR showed a two–three log improvement compared to conventional qPCR, and 24 of 32 samples negative with qPCR resulted positive with r-qPCR and/or dPCR [10]. These preliminary data were subsequently confirmed by other reports [12,96,97]. In particular, Wang and colleagues tested 10 CML patients in FU with a MR4.5, and ddPCR revealed positivity three months earlier than by qPCR in four patients. Therefore, ddPCR seems to be more sensitive than qPCR, allowing better conversion of ddPCR results into International Standard qPCR data, to achieve a deeper molecular biology-based stratification of *BCR-ABL1* MRD [96].

Another important aspect on which dPCR studies have been focused is the discontinuation of tyrosine kinase inhibitors (TKI) therapy for CML patients’ treatment, associated with the prediction of relapse. Some studies have judged dPCR to be a method that could help to prognosticate relapse after TKI discontinuation earlier than qPCR [98,99,100]. Colafigli et al., for example, in prospective longitudinal monitoring after TKI discontinuation, showed that ddPCR anticipated the relapse by three months. ddPCR is a precise molecular approach that, as compared to qPCR, seems to be superior as regards the quantification of low levels of residual disease in patients who have already obtained a stable response. It may thus help to curb the failure of treatment discontinuation [100].

Lastly, Cumbo et al. reported a study of MRD monitoring in CML with a DNA-based assay to enhance the sensitivity of detection of *BCR-ABL1* positive cells, in detecting the *BCR-ABL1* genomic rearrangement. For the establishment of the *BCR-ABL1* junction sequence, Cumbo et al. proposed two alternative approaches: the first one consisted of a first fluorescence in situ hybridization (FISH)-based step followed by SS; the second one employed MinION, a nanopore third generation sequencing technology that the research group consolidated [101,102,103,104,105]. Once the *BCR-ABL1* genomic junction had been defined, ddPCR was employed for patient “personalized monitoring” [106].

MRD is the strongest prognostic factor in Acute Lymphoblastic Leukemia (ALL), able to predict response to treatment and risk of relapse [107]. However, to date, only few studies have explored the utility of dPCR application in ALL MRD assessment. The reasons for this paucity could lie in the assumption that qPCR is a consolidated tool for MRD monitoring, despite the demonstration of a superior sensitivity of dPCR. In fact, qPCR is not always able to precisely define the amount of residual disease, frequently classifying samples with a very low MRD level as “positive not-quantifiable” (PNQ), a definition that is poorly interpretable. Indeed, another important limitation of qPCR is the need for a standard curve based on diagnostic DNA. In 2016, Della Starza and colleagues compared the two quantification techniques in the analysis of 50 ALL cases, reporting a sensitivity and accuracy for ddPCR that was at least comparable to those of qPCR [108]. Subsequently, in 2019, the same research group focused attention on FU adult ALL samples with a very low disease load (qPCR MRD levels ≤10^−4^), using ddPCR and NGS for analyzing *immunoglobulin/T-cell receptor* gene rearrangements as molecular markers [109]. This comparison showed a concordance rate of 57% (13/23) for qPCR/ddPCR and 52% (12/23) for qPCR/NGS; indeed, ddPCR and NGS also identified positivity in samples with a very low disease burden, yielding concordant MRD results in 87% of samples. Moreover, ddPCR/NGS analysis significantly reduced the number of PNQ samples compared to qPCR, increasing the number of quantifiable samples and helping to identify three relapses in patients that resulted PNQ/NEG through qPCR, as already previously reported [110]. These data demonstrated that in ALL, dPCR and NGS could more precisely stratify samples with very low MRD, for which qPCR is unable to detect or to quantify the disease burden [109]. As regards expression analysis, two studies employed dPCR for ALL MRD monitoring, analyzing Philadelphia-positive (Ph+) cases with the *BCR-ABL1 p190* fusion transcript (m-bcr), which account for 20%–30% of adult ALLs [111,112,113]. In the study reported in 2014 by Iacobucci et al., 60 *BCR-ABL1+* ALL samples in hematologic and cytogenetic remission were analyzed using the microfluidic dPCR approach (Biomark system from Fluidigm) [114]. They demonstrated that the assay was able to detect until a single copy of *BCR-ABL1* transcript, with results at least comparable with those obtained with the conventional qPCR test. However, as compared to the dPCR technologies available today, this system was less sensitive and accurate, as only about 9000 partitions could be generated and the total assay volume was limited to 8 µl; indeed, for each sample the total amount of analyzed RNA was about 20ng [23,114,115]. More recently, Coccaro et al. conducted a second study, this time performing ddPCR to define its possible predictive molecular value during *BCR-ABL1+* ALL FU [116]. With ddPCR, the evaluable sample volume is 20 ul, and partitioning reaches 20,000 droplets, allowing for higher accuracy and sensitivity [23]. FU samples showed that the p190 ddPCR assay was able to quantify very small disease levels by loading a high quantity of cDNA (up to 750ng per well) in different wells and combining the counts from multiple replicates. Of note, scaling up the ddPCR reaction to 750 ng of cDNA did not have a negative impact on the reaction performance, as data from serial dilutions performed loading such amount of cDNA showed remarkable linearity, reliability, and a precision of up to 0.001%. Comparison of the results obtained with conventional PCR and ddPCR in 117 FU samples showed discordant results in 27% cases, and further analysis through qPCR showed 19 ddPCR positive samples with a low tumor burden that was negative to PCR, failing to provide quantitative results in 63% of cases, classifying three samples as negative and nine as PNQ. This emphasizes the concept, that for qPCR, there is a gap between the sensitivity and the quantitative range. This is a critical limitation, as borderline cases that fall in the range of inadequate quantification cannot be classified though qPCR, whereas this can be carried out with ddPCR. The demonstrated higher sensitivity of the digital *p190* assay as regards PCR and/or qPCR was, indeed, demonstrated to be able to predict molecular relapse, offering a timely advantage for patient management. In fact, it may allow a rapid change of therapy before hematological relapse [117,118,119,120,121,122] and thus improve the chances of preventing disease progression [116].

## 4. dPCR and Transplantation

Patients with acute myeloid leukemia benefit from allogeneic hematopoietic stem cell transplantation (allo-HSCT), which is an established consolidation therapy. However, too often, they suffer relapse after allo-HSCT, that confers poor prognosis, and this remains a major clinical challenge. In this context, the early identification of patients at high risk of relapse, and thus the detection of MRD, is essential, as well as assessment of the successful outcome of the transplant procedure. Therefore, several groups have tested the feasibility of the dPCR method for these purposes (Figure 1).

dPCR was employed for chimerism analysis in transplanted patients, the term “mixed chimerism” (MC) meaning the condition of a mixture of genetic profiles resulting after allo-HSCT. A series of studies demonstrated the power of dPCR as a tool for clinical chimerism analysis as compared to established methods based upon Indel and short tandem repeats (STRs) detection [123,124,125,126,127,128]. George et al., in 2013, were the first to develop a dPCR-based method for quantifying chimerism. They demonstrated, for dPCR, a higher accurate quantification in comparison to qPCR, down to 0.01% and with the potential to go even lower [123]. After this, Stahl et al. compared dPCR to qPCR, whose proved sensitivity for chimerism analysis was one patient cell in >1000 donor cells, presenting, however, limited resolution power in the state of mixed chimerism (e.g., >10% patient cells) [124]. They screened more than 10 dPCR assays detecting Indel polymorphisms or Y-chromosome sequences, testing them on artificial cell mixtures and patient samples. They demonstrated that dPCR is able to perform exact quantification of chimerism over several orders of magnitude and confirmed its high reproducibility, especially in the “difficult” range of mixed chimerism. On patient samples, dPCR showed excellent performance (with a sensitivity of 0.03% analyzing 65ng of DNA), in comparison with qPCR and short-tandem repeat PCR methods [124]. Further, Waterhouse et al. employed the ddPCR technique to monitor MRD and chimerism simultaneously, fully evaluating the molecular remission status of transplanted patients [128]. The authors analyzed 764 samples from 70 patients after HSCT, showing MC in 219 samples from 37 patients, with a 4.3% mean percentage of host-derived DNA in positive samples, while in 15 patients, MRD positivity and/or increased WT1 expression was evident, using *NPM1* (Type *A, B, K*), *DNMT3A* (*R882H*), *MLL-PTD*, *IDH1* (*R132H*) and *KRAS* (*G12S*) as MRD markers. Indeed, in 15 patients, increasing MC values and positivity for an MRD marker, increased WT1 expression or both, were detected. Furthermore, they determined whether MC or MRD positivity was the first to be detected, highlighting that MC was observed first in six patients and MRD positivity in two patients; in the remaining seven patients they were detected simultaneously [128].

dPCR has also been demonstrated to be useful for monitoring platelet engraftment after allo-HSCT. It is normally examined by daily platelet counts, but any necessary platelet transfusions performed in the patient can obscure the detection of platelet engraftment. Using ddPCR, Doescher at al. drew up an experimental assay based on mitochondrial DNA isolated from platelets in order to quantify circulating platelets derived from the stem cell graft, distinguishing them from transfused single-donor apheresis platelets [129]. Consecutive daily PB samples from day 7 to day 20 after transplantation were collected from 22 patients. The authors defined platelet engraftment as the third of at least three consecutive days of increasing levels exceeding 1000/mL of endogenous platelets. They assessed platelet counts according to the engraftment criteria of the Center for International Blood and Marrow Transplant Research (CIBMTR) [130] and the European Society for Blood and Marrow Transplantation (EBMT) [131]. When analyzing the results in detail, they found evidence that the moment of platelet engraftment based on the EBMT criteria corresponded to the ddPCR observation of transplantation-derived platelets, confirming that this ddPCR test is a sensitive method for monitoring platelet engraftment without interference due to platelet transfusions [129].

In several other studies, dPCR was applied for MRD detection in patients undergoing allo-HSCT. Bill et al. assessed mutated-*NPM1* MRD-positivity with ddPCR in pre-transplant samples from 51 *NPM1*-mutated AML patients [132]. They observed that positive patients had a higher cumulative incidence of relapse and shorter OS, and these conclusions were also the same after restricting the analyses to patients receiving non-myeloablative conditioning. Thus, the presence of MRD positivity for mutated-*NPM1* confers a worse prognosis, independently of other known prognostic markers [132]. 

In the work conducted by Brambati et al., ddPCR experiments were performed utilizing *DNMT3A*, *IDH1*, and *IDH2* as MRD markers; these are considered disease founder mutations and their frequent alteration, in particular in *DNMT3A*, seems to occur very early during leukemogenesis and thus are likely shared by all disease subclones and steadily preserved until the eventual relapse [133,134,135,136,137]. They studied 30 transplanted patients, showing positivity for at least one mutation; for 17 of the patients they analyzed FU samples harvested at post-transplant hematologic remission. Nine of the 17 patients relapsed post-transplantation and in seven of them ddPCR positivity was evident at one pre-relapse time point at least, highlighting a 78% ddPCR sensitivity value in predicting relapse. Moreover, the BM sample immediately prior to the relapse was ddPCR positive in six patients, with a median time from first positivity to relapse of 60 days. When the ddPCR results on *DNMT3A* and *IDH1/2* were compared with qPCR analysis for *NPM1* mutations, for the WT1 transcript, and for host-specific hematopoietic chimerism, the data seemed be largely concordant. However, as these mutation-specific assays are informative only for specific patients, and the molecular markers might be lost due to molecular disease evolution, the optimal way to tackle MRD is by a multi-target patient-specific approach [137]. 

As regards expression analysis, ddPCR was employed in two studies to explore the expression of the *BAALC* [138] and *MN1* genes [139]. For both genes, high levels of expression are frequently found in AML at diagnosis, and these have been demonstrated to be associated with an adverse outcome and hence are useful as markers for residual disease following therapy [140,141,142,143,144,145,146,147,148,149,150]. ddPCR experiments showed that cases with high pre-HSCT *BAALC* copy numbers were strongly associated with a higher cumulative incidence of relapse within 100 days after HSCT, and shorter OS [138]. Similarly, high *MN1* gene expression was associated with a higher cumulative incidence of relapse after HSCT and with a shorter time to relapse. Indeed, AML cases with the highest level of *MN1* gene expression presented the highest risk of relapse [139]. Thus, the study of pre-HSCT *BAALC* and *MN1* expression using ddPCR, together with analysis of the *NPM1* mutation and *WT1* expression, could be a valid approach to identify AML patients at high risk of early relapse after HSCT. Further prospective studies are needed to confirm their prognostic value [138,139]. 

Recently, Fehse et al. employed ddPCR also to detect and quantify transgenic *CAR-T* (*chimeric antigen receptor T*) cells transfused in vivo in refractory/relapsed Diffuse Large B-Cell Lymphomas (DLBCL) and primary mediastinal large B cell lymphoma (PMBCL) patients treated with axicabtagene ciloleucel (Axi-cel) *CD19-CAR-T* cells [151]. For this purpose, different combinations of primers and dual-labeled hydrolysis probes mapping in various *CAR* regions were designed and tested in duplex reactions, using a reference gene to concomitantly assess cell numbers. Three of these designed assays were demonstrated to be specific and reproducible, with an almost perfect correlation between the *CAR* target and the reference, and with a LOD of one single *CAR*-transduced cell, corresponding to a sensitivity of 0.01% for 100 ng genomic DNA. In vivo application to monitor Axi-cel *CAR-T* numbers in patients showed *CAR-T* fluctuations, as expected, and the median peak value of Axi-cel *CAR-T* cells was 11.2/mL at 11.3 days. Indeed, they demonstrated good cell expansion with clinical responses in four of the five patients (two CR and two partial responses) on day 30, demonstrating the utility of the ddPCR assay in the clinical management of these patients [151].

## 5. Other dPCR Applications and Evolution

Frequently, dPCR has been employed as a validation tool to corroborate data obtained with other technologies (Figure 1). In research conducted in 2019, Jespersen et al. performed ddPCR experiments to corroborate data regarding alternative splice variants of a series of candidate genes identified through exon array analysis, in order to demonstrate that specific alternatively spliced *NOTCH3* isoforms display a prognostic and predictive biomarker potential in Germinal Center B-cell like (GCB) DLBCL [152]. Beheshti et al. used ddPCR for the quantification of a microRNA signature composed of 10 circulating miRNAs that strongly involved *JUN* and *MYC* oncogenic signaling, and that they hypothesized them to be age-dependent and to influence DLBCL development in the murine model [153]. ddPCR was used also to validate data obtained from microarray analysis in studies on AML [43] and CLL [70].

Often, the dPCR and the NGS are used together [154,155]. Apart from showing increased sensitivity and specificity, the two technologies present some peculiar characteristics: NGS is able to detect unknown and/or multiple genetic modifications even if it is labor- and time-intensive and requires informatic support; on the other hand, dPCR is time-saving, cheaper and easy to perform, but it requires prior knowledge of the molecular alterations to be identified, and also has limited multiplexing possibilities [156,157,158]. Considering these features, the two methods are frequently employed in combination, especially for liquid biopsies analyses where NGS is generally exploited in the discovery step and dPCR in the validation step [159,160,161,162]. For example, Mithraprabhu et al. carried out ddPCR experiments to validate the mutations found in MM patients in a study using NGS to compare analyses of plasma (PL)-derived ctDNA versus BM biopsy. Indeed, through ddPCR, they tracked and quantitated specific clones in sequential ctDNA obtained across longitudinal PL samples, evidencing fluctuations of clone fractional abundances depending on the disease status [163]. These data promoted the concept that the utility of analyzing, with dPCR and NGS, ctDNA besides BM biopsy consists in deeper molecular characterization and therapeutic monitoring of MM, tracking the emergence of relapsing clones that could be attacked by implementing therapy changes [163].

Furthermore, a series of studies have employed dPCR for other applications (Figure 1). A convenient use consists in the detection and quantification of genetic biomarkers lacking laboratory standards (for example, gene- and transcript-fusions, or rare transcripts) for which conventional methods such as qPCR cannot be employed [164,165,166,167,168,169,170]. For example, Coccaro et al. recently developed a ddPCR assay for the *BCR-FGFR1* fusion transcript for MRD monitoring in a rare case of ALL with t(8;22)/*BCR-FGFR1* gene fusion, for which standardized methods are lacking demonstrating that the ddPCR assay was capable of predicting hematologic relapse with a higher sensitivity than the classical PCR method [164].

dPCR is useful for discriminating transcript variants: some researchers employed the method in a study on CML to establish qPCR efficiency in identifying *BCR-ABL1* transcript variants. Kjaer et al. analyzed 219 CML patients with either the *e13a2* (n = 113) or *e14a2* (n = 106) variant, enrolled at three Danish diagnostic centers, and the data obtained suggested that the qPCR assay may underestimate the *e14a2* variant compared to the *e13a2* variant, since the analysis of qPCR vs. ddPCR values revealed a significant average difference in the bias between the variants (*e3a2/e14a2*) at the three centers. These data were concomitantly confirmed by Bernardi et al. [171,172]. ddPCR was also employed as an alternative to FISH in CML for breakpoint characterization, noting, apart from improved specificity and sensitivity, the possibility of identifying variant translocation patterns, including derivative chromosome 9 deletions [173].

Another potential application of dPCR is DNA methylation analysis, which has been explored in several studies of malignancies [155]. In the hematologic field, Orsini et al. recently proposed a ddPCR method to investigate Alu differential methylation for the use in profiling patients affected by hematologic malignancies for diagnostic/prognostic purposes [174].

Moreover, some authors have recently tried to overcome some limitations of the dPCR approach, such as its employment as a discovery tool, that is limited by the need for a priori knowledge of the mutation to be detected; this limitation restricted the number of alterations screened per reaction, simultaneously increasing the number of probes employed to analyze each known variant allele. In order to face this challenge, an alternative dPCR approach, called drop-off dPCR, was developed; it requires only a single pair of probes to find and quantify all genetic alterations in a targeted region in a single reaction. In this system, the FAM-labeled probe recognizes a reference sequence distant from the target but within the same amplicon, and the HEX-labeled probe binds the target wild-type sequence [175,176,177,178,179]. Therefore, the mutated sequences only generate the FAM signal, while the wild-type sequences display both FAM and HEX signals. For drop-off ddPCR, comparable sensitivity to conventional ddPCR assays has been demonstrated, with the advantage of being cost-, time- and sample-effective, as it enables the detection of a greater number of mutations in a single reaction. Recently, a research team applied this approach when studying *IDH2* gene mutations in AML patients at diagnosis and follow-up during treatment, obtaining hopeful results [75]. Alcaide et al., instead, focused their efforts using this method for the development of a ddPCR assay for the detection and quantification of mutations affecting hotspot residues *Y641* of *EZH2* and *D419* of *STAT6* in fresh tumor, FFPE, and liquid biopsies in B-cell non-Hodgkin lymphomas, including DLBCL, FL, and lymphoplasmacytic lymphoma. Indeed, they tested multiplexing of these probes with two others designed for two additional lymphoma-related hotspots (*L265P* of *MYD88* and *I290R* of *CCND3*), allowing the simultaneous detection of distinct mutations [180]. Finally, they designed “inverted” ddPCR assays, in which they used matching probes on *EZH2* and *STAT6* wild-type alleles, capable of highlighting the presence of multiple mutations [180].

Another alternative application of ddPCR is Somatic Mutation Allelic Ratio Test ddPCR (SMART-ddPCR). In 2015, De Smith et al. employed this method to assess tumor preferential allelic imbalance (PAI), a phenomenon by which a tumor selects single nucleotide polymorphism (SNP) risk alleles and the associated somatic copy number alterations in tumor DNA. This method was successfully applied to test the hypothesis that heritable SNPs associated with childhood ALL may exhibit tumor PAI, comparing results with those obtained from multiplex ligation-dependent probe amplification (MLPA) [181].

## 6. Conclusions

Nowadays, when we talk about the personalized treatment of cancer, the need arises to use the most reliable technology, which should be robust and advantageous in terms of the savings of economic resources and time, as well as of ease of use.

In the hematologic field, the reported data show dPCR as a rapid and reliable technology for obtaining the absolute quantification of a molecular target at disease diagnosis, during MRD assessment, as well as after HSCT in routine clinical laboratories, without any need for calibrators, standard curve, or laboratory conversion factors. The main advantages of this technology are the high precise and reliable quantification and the excellent reproducibility, as compared to qPCR. As regards the search of somatic mutations, several findings corroborated the feasibility and utility of dPCR, particularly in the context of liquid biopsy as an alternative to BM or tissue biopsy analysis, for example, in the case of cHL and WM. In the context of MRD evaluation, there is a broad consensus that the observation of a complete molecular response (CMR) shortly after the beginning of treatment, such as chemotherapy, plus a TKI for Ph+ ALL, for example, should be interpreted as a strong prognostic factor that guides the choice of transplant option [120]. From this point of view, the ability to delve deeper and deeper into the MRD positivity issue is a crucial link between patient management and the selection of therapy. In the case of ALL, for example, failure to achieve CMR could drive the decision to perform allo-HSCT, whereas the evidence of stable CMR could indicate the lack of need for transplantation, being associated with an improved survival [119,120,121,182,183]. dPCR also represents a good promise for monitoring transplantation assessment, studying chimerism and determining quantification of CAR-T cells in transplanted patients. Nevertheless, the findings regarding the dPCR sensitivity in comparison to qPCR are still discordant, as it seems to depend on the kind of analyzed nucleic acid, as well as on the assay set up. Saturation tests, loading a high amount of template, showed good performance for ddPCR assays for *p210* and *p190 BCR-ABL1* transcripts [12,116]. However, these studies performed singleplex reactions, analyzing the reference gene in separate wells in order to avoid positivity of all individual reactions. In addition, the analyses on DNA are a priori limited by the amount of gDNA that can be load per reaction because of its viscosity, which can affect reaction performance. Thus, for this kind of comparison, further studies are needed to settle the matter.

Regarding its relation with NGS, dPCR is employed for the accurate determination of DNA and RNA throughout NGS workflows, including the final library quantification. Indeed, dPCR is faster and cheaper than conventional NGS, with even higher sensitivity, lower probability of errors, need of less DNA input, and without bioinformatics pipelines [17]. These features seem to render dPCR superior for MRD monitoring; accordingly, optimized NGS methods have been explored in the last few years in order to solve limitations in terms of sensitivity and specificity [17,155]. Looking at a clinical translation, providing complementary information, these two technologies could ideally work in synergy, where NGS is employed for the discovery step and dPCR for the validation and monitoring.

The choice of the analysis technique should be that of the most precise and sensitive method. These needs justify the efforts spent on the development of the digital technology, considering the high expectations regarding sensitivity and specificity. As regards the hematologic malignancies, several studies conducted in the last few years confirmed the good performance of dPCR. However, we are still far from the goal of stable implementation of this technology in everyday use. The reasons for this probably lie in a reluctance to abandon consolidated procedures, but this is not the only reason. For some kinds of analysis, such as MRD study in CML and ALL *BCR-ABL1+*, qPCR one-step kits are available, which allow for the analysis of a high number of samples. Simple as the experimental dPCR protocols are, they are still slightly more laborious than qPCR one-step procedures. A solution might be automation, which could be time-saving and have the advantages of greater sensitivity and precision. However, it is unlikely that this will be the key for the worldwide diffusion of dPCR. This technology is still relatively highly expensive in regards equipment and single reactions, so nowadays, qPCR still enjoys greater diffusion in clinical practice, remaining the gold standard for several types of analyses. Only standardization programs will give the proper launch for including dPCR in everyday practice. Despite the clear advantages of this technology, there is still a lack of studies of large cohorts of patients and standardization of procedures. For this purpose, a large standardization study is currently ongoing within the European Scientific Foundation for Laboratory Hemato Oncology (ESLHO). Accordingly, in the future, it will be necessary to schedule further controlled clinical trials and multilaboratory standardization programs to evaluate dPCR as an outcome predictor tool.

## Abbreviations

AITL	angioimmunoblastic T-cell lymphoma
Allo-HSCT	allogeneic-HSCT
AMLs	Acute Myeloid Leukemias
APL	acute promyelocytic leukemia
BM	bone marrow
CAR-T	chimeric antigen receptor T
CBF	core binding factor
cfDNA	cell-free DNA
cHL	Classical Hodgkin lymphoma
CLL	Chronic Lymphocytic Leukemia
CML	Chronic myeloid leukemia
CNS	central nervous system
CR	complete remission
DLBCL	Diffuse Large B-Cell Lymphomas
dPCR	digital PCR
ddPCR	droplet digital PCR
ET	essential thrombocythemia
FA	fractional abundance
FFPE	formalin-fixed paraffin-embedded
FISH	fluorescence in situ hybridization
FL	Follicular Lymphoma
FLA	Fragment length analysis
FU	follow-up
HSCT	hematopoietic stem cell transplantation
IgH	immunoglobulin heavy chain
MC	mixed chimerism
MCL	Mantle Cell Lymphoma
MF	Myelofibrosis
MLPA	Multiplex ligation-dependent probe amplification
MM	Multiple Myeloma
MRD	Minimal Residual Disease
NGS	Next-Generation Sequencing
OS	Overall survival
PAI	preferential allelic imbalance
PB	peripheral blood
PC	plasma cells
PCNSL	central nervous system lymphomas
PFS	progression-free survival
Ph- MPNs	Philadelphia negative chronic Myeloproliferative Neoplasms
PMBCL	primary mediastinal large B cell lymphoma
PNA-LNA	peptide nucleic acid-locked nucleic acid
PNQ	positive not-quantifiable
PTCL	peripheral T-cell lymphoma
PV	polycythemia vera
qPCR	Real-time Quantitative PCR
SMART-ddPCR	Somatic Mutation Allelic Ratio Test ddPCR
SNP	single nucleotide polymorphism
SS	sanger sequencing
STRs	short tandem repeats
TDS	targeted deep sequencing
TKI	tyrosine kinase inhibitors
VAF	variant allele fractions
WM	Waldenström Macroglobulinemia
